# The relation between physical joint examination and MRI-depicted inflammation of metatarsophalangeal joints in early arthritis

**DOI:** 10.1186/s13075-020-02162-7

**Published:** 2020-04-03

**Authors:** Yousra J. Dakkak, Aleid C. Boer, Debbie M. Boeters, Ellis Niemantsverdriet, Monique Reijnierse, Annette H. M. van der Helm-van Mil

**Affiliations:** 1 Department of Rheumatology, Leiden University Medical Center, P.O. Box 9600, 2300 RC Leiden, the Netherlands; 2 Department of Radiology, Leiden University Medical Center, Leiden, the Netherlands

**Keywords:** Rheumatoid arthritis, Early arthritis, Physical joint examination, Magnetic resonance imaging, Foot

## Abstract

**Background:**

The relationship between physical joint examination (PE) and MRI-detected inflammation in early inflammatory arthritis has mostly been studied in the hands. Physical examination of MTP joints is considered difficult, and for these joints, this relationship is unknown. Therefore, we studied the concordance of PE with MRI inflammation in MTP joints. Metacarpophalangeal (MCP) joints were included for comparison.

**Methods:**

One thousand seven hundred fifty-nine MTP(2–5) and 1750 MCP(2–5) joints of 441 consecutive patients with early arthritis underwent PE (for joint swelling) and MRI, all evaluated by two assessors. MRI was scored for synovitis, tenosynovitis, and osteitis (summed MRI inflammation). Synovial intermetatarsal bursae may enlarge upon inflammation and become palpable and were therefore also assessed. Analyses (frequencies, GEE) were performed on joint level.

**Results:**

PE and MRI were concordant in 79% of MTP joints. Of 1606 non-swollen MTP joints, 83% showed no MRI inflammation and 17% showed subclinical MRI inflammation. Of 153 swollen MTP joints, 48% had MRI inflammation and 52% (79 MTP joints) did not. Of these 79 swollen MTP joints without MRI inflammation, 31 showed intermetatarsal bursitis and 48 joints had none of these MRI abnormalities (this concerned 31% of swollen MTP joints). MTP swelling was statistically independently associated with tenosynovitis (OR 2.21, 95% CI 1.1–4.3) and intermetatarsal bursitis (OR 2.91, 95% CI 1.8–4.8).

MTP joints showed subclinical inflammation less often than MCP joints (17% vs. 34%, *P* < 0.001). Swollen MTP joints showed MRI inflammation less often than swollen MCP joints (48% vs. 88%, *P* < 0.001).

**Conclusions:**

The absence of swelling of MTP joints in early arthritis is mostly accompanied by the absence of MRI-detected inflammation. Swollen MTP joints are, in addition to synovitis, also explained by tenosynovitis and intermetatarsal bursitis and partly unexplained by MRI. Their clinical relevance must be determined in longitudinal studies.

## Introduction

Rheumatoid arthritis (RA) is characterized by chronic joint inflammation, especially of the small joints [1, 2]. In recent-onset RA, the metatarsophalangeal (MTP) joints are often involved, which can generate walking disabilities [3–5]. Physical joint examination (PE) for joint swelling upon palpation is a crucial element in the assessment of the clinical status of RA patients. It is however known that PE of the MTP joints can be difficult [6].

Magnetic resonance imaging (MRI) of the hands is more sensitive than PE in detecting inflammation, and its use has been recommended in the management of RA [7]. However, the relation between inflammation detected at PE and with MRI at the MTP joints is unclear. Studies on this relationship focused on the wrist and metacarpophalangeal (MCP) joints [8–10]. Only two studies investigated the feet; the first study was relatively small (*n* = 40) and was performed in patients receiving disease-modifying anti-rheumatic drugs (DMARDs) and not in untreated patients [11]. The second study did not use contrast enhancement [10], limiting the conclusions that could be drawn as contrast enhancement is required to sensitively depict synovitis [12]. Also, neither evaluated tenosynovitis at the MTP joints.

In addition to the classic inflammatory features (synovitis, tenosynovitis, and osteitis), intermetatarsal bursitis (IMB) is also prevalent in inflammatory arthritis [13, 14]. Interestingly, intermetatarsal bursae in the forefoot have a synovial lining and are located between the metatarsal heads immediately above the deep transverse metatarsal ligament; a schematic illustration of the forefoot with the anatomic position of these bursae is given in Additional file 1: Figure S1 [15]. Inflammation may lead to enlargement and dorsal protrusion of the bursae, above the metatarsal heads [16, 17]. Theoretically, IMB can therefore be palpable. IMB have not previously been studied in respect to swelling upon PE and were therefore also included in this study.

The currently available literature does not give a fulfilling and thorough representation of the relationship between PE and MRI of the MTP joints. To increase the understanding of this relationship, we performed a large cross-sectional study in early arthritis patients and assessed the concordance of arthritis upon PE and MRI-detected inflammation at the MTP joints. MCP joints were included for comparison.

## Methods

### Participants

Four hundred forty-seven consecutive patients newly presenting with clinically confirmed arthritis were included in the Leiden Early Arthritis Cohort between June 2013 and March 2016. Included patients had a symptom duration < 2 years and were naïve to DMARDs [18]. At baseline, PE of joints was performed, serum samples were taken, and an MRI was performed before DMARD initiation. The median time between PE and MRI was 7 days (95% confidence interval (CI) 3–14 days).

MRIs of 6 patients were excluded because of inhomogeneous fat suppression. Of the remaining 441 patients, 157 were classified as RA, defined as a clinical diagnosis plus fulfillment of the 2010 RA criteria during the first year of follow-up [1]. The remaining 284 patients received alternative diagnoses and are presented in Table 1.

**Table 1:** Baseline characteristics of patients that presented with early arthritis

	Patients (*n* = 441)
Age, mean (SD)	57 (16)
Female, *n* (%)	267 (61)
Symptom duration, in weeks, median (IQR)	9 (4–27)
66-Swollen joint count, median (IQR)	3 (1–7)
CRP (mg/L), median (IQR)	7 (3–20)
ACPA positive, *n* (%)	156 (37)
Diagnosis, *n* (%)	
Rheumatoid arthritis (RA)	157 (36)
Unclassified arthritis	148 (33)
Psoriatic arthritis or pondyloarthritis	45 (10)
Inflammatory osteoarthritis	23 (5)
Reactive arthritis	7 (2)
RS3PE	12 (3)
SLE and MCTD	5 (1)
Other diagnoses	44 (10)

*SD* standard deviation, *IQR* interquartile range, *CRP* C-reactive protein, *ACPA* anti-citrullinated peptide antigen, *RA* rheumatoid arthritis according to clinical diagnosis and 2010 criteria during the first year of follow-up, *RS3PE* remitting seronegative symmetrical synovitis with pitting edema, *SLE* systemic lupus erythematosus, *MCTD* mixed connective tissue disease. The 66-swollen joint count was assessed

### Physical joint examination

The presence of joint swelling upon PE was evaluated by two independent assessors, a rheumatologist and a trained research nurse, with the patient in supine position on the exam table. Research nurses held regular consensus exercises for swollen joint assessment led by a rheumatologist to maintain high interobserver agreement. In this study, PE was considered positive for joint swelling if both assessors independently scored its presence in the same joint.

### MRI scanning and scoring

Unilateral scans were acquired of the MTP and MCP joints of the most painful side, or the dominant side in case of equally severe symptoms, on a 1.5-T extremity MRI (General Electric, WI, USA). Gadolinium contrast was administered intravenously. Sequences acquired were pre-contrast coronal T1-weighted fast spin echo (T1), post-contrast coronal and axial T1-weighted fast spin echo with frequency-selective fat saturation (T1Gd) of MCP joints, and post-contrast coronal and axial T1 of the MTP joints. For a more detailed description of the MRI protocol, see Additional file 1: Supplementary Methods 1.

Synovitis and osteitis of MTP(2–5) and MCP(2–5) joints were scored in line with the RAMRIS [19], with the exception that osteitis was assessed on a contrast-enhanced T1-weighted fat-suppressed sequence, as its use for depicting osteitis is recommended by the European Society of Musculoskeletal Radiology (ESSR) and previous studies have demonstrated that it has a strong correlation with the T2-weighted fat-suppressed sequence that is advised by the RAMRIS [20–23]. For tenosynovitis, the score as described by Havaardsholm et al. was applied to the extensor and flexor tendons of the MTP and MCP joints [24]. Additional information on the method of scoring is provided in Additional file 1: Supplementary Methods 2. Each MRI was scored by two independent readers, blinded to clinical data. Interreader and intrareader intraclass correlation coefficients (ICCs) were generally ≥ 0.90 and were published previously [25]. MRI-detected inflammation was positive per joint if synovitis, tenosynovitis, and/or osteitis were scored as ≥ 1 by both readers independently.

Scoring of IMB was done at intermetatarsal spaces by two readers blinded to clinical data adjusted from Cherry et al. as is described below [26]. The first reader (MR) was a trained musculoskeletal radiologist with 23 years of experience, and the second reader (YD) was an MD who had scored > 400 MRIs according to the RAMRIS method during a training period of 12 months prior to evaluating the MRIs that are part of this study. IMB was defined as a contrast-enhanced lesion with or without rim enhancement that was located between the metatarsal heads above the deep transverse metatarsal ligament that could protrude at the dorsal side of the metatarsal heads, as is illustrated in Additional file 1: Figure S1. IMB was scored as present when visible on two consecutive slices in both axial and coronal planes. As this is not a validated scoring method, scoring was done in consensus: IMB at MTP joints was considered positive if found present by both readers in consensus.

As mentioned, MCP joints were included as comparison. Synovitis, tenosynovitis, and osteitis were scored as in the MTP joints. Bursae between MCP joints are unknown in the literature and were not scored routinely. However, literature can be incomplete, and therefore, in the subgroup of joints that were swollen but MRI negative, one reader explored whether there could be intermetacarpal bursitis. This revealed no signs of local bursitis; therefore, further efforts to search for bursitis at the MCP joints were deemed unnecessary.

### Analyses

Analyses were performed separately for MTP and for MCP joints: the main analysis was of MTP joints, MCP joints were included for comparison. Frequency of concordance/discordance of PE and MRI was evaluated on joint level. The MRI values of 19 out of 3528 joints (5 MTP joints and 14 MCP joints) were missing, as these joints could not be scored due to movement artifacts or inhomogeneous fat suppression. None of these 5 MTP joints was swollen upon PE; 5 out of the 14 MCP joints were swollen. Joint-level analyses were performed; therefore, these values were not imputed.

For the joint-level analyses of PE and IMB, the nearby location was considered, e.g., when studying PE of MTP3, IMB in the second and third intermetatarsal spaces were considered. Generalized estimating equations (GEE) were done to assess the association of MRI features with clinical swelling, as this model takes into account that in every patient multiple joints were assessed.

To further support the results of our main findings in MTP joints, 3 sub-analyses were performed. First, the relation for PE with MRI-detected inflammation was evaluated in the subgroup of RA patients. Next, we speculated that osteitis and in addition flexor tenosynovitis may not be palpable, as PE is performed at the dorsal side of MTP joints. Therefore, the frequency of concordance/discordance was studied excluding flexor tenosynovitis and osteitis from the definition of MRI inflammation. Finally, non-swollen joints may be tender as a sign of ‘subclinical’ inflammation; to study this, analyses were repeated in joints that were tender and swollen joints were excluded from this analysis.

## Results

### Baseline characteristics

**Fig. 1:**
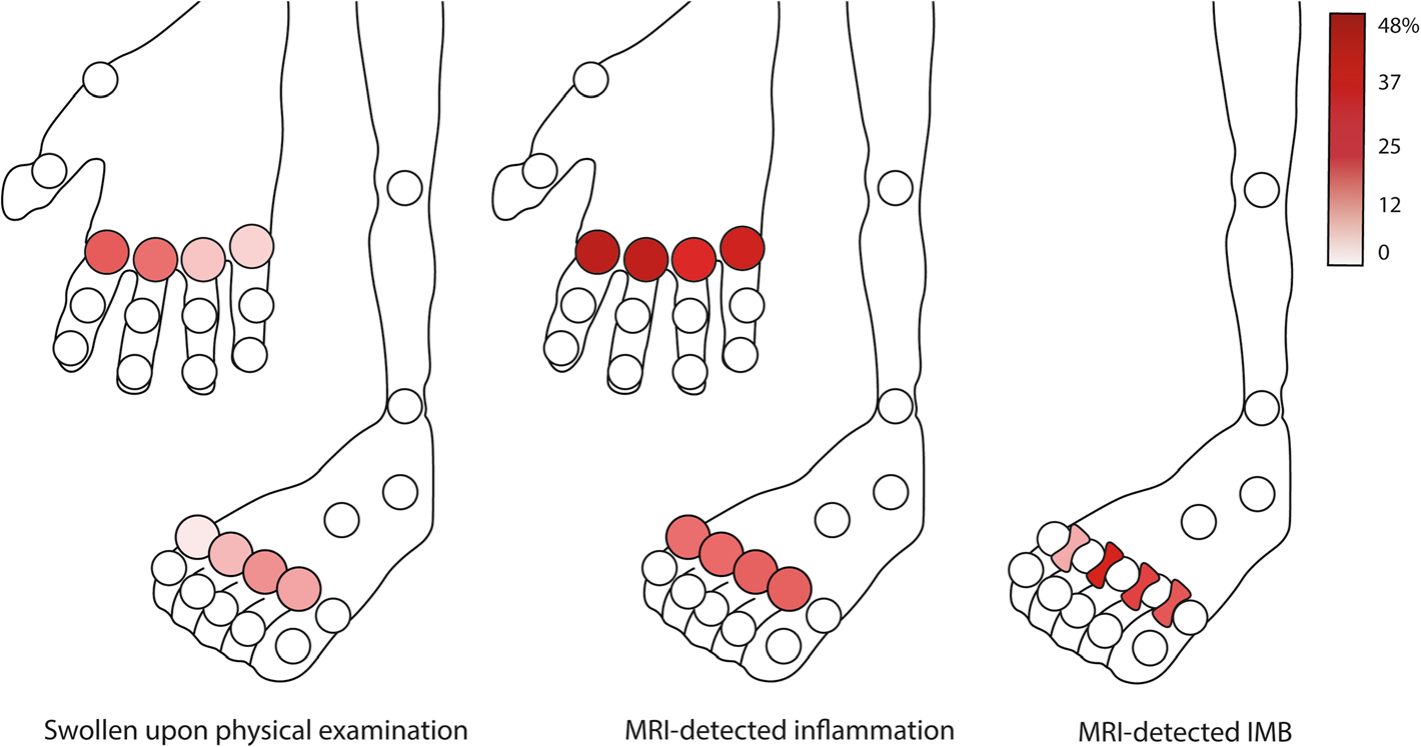
Frequency of a swollen joint upon physical joint examination, MRI-detected inflammation, and intermetatarsal bursitis (IMB) per location for MTP(2–5) and MCP(2–5) joints in consecutive patients presenting with early arthritis. Physical joint examination was positive if two assessors independently scored the joint as swollen. MRI-detected inflammation was positive per joint if synovitis, tenosynovitis, and/or osteitis were scored as present by two readers independently

Baseline characteristics of the 441 patients are presented in Table 1. Seventy-three patients (17%) had ≥ 1 swollen MTP joint, and MTP2 and MTP3 were most often swollen (Fig. 1). One hundred seventy-six patients (40%) had MRI-detected inflammation at least one MTP joint.

### Concordance and discordance of physical joint examination and MRI

**Fig. 2:**
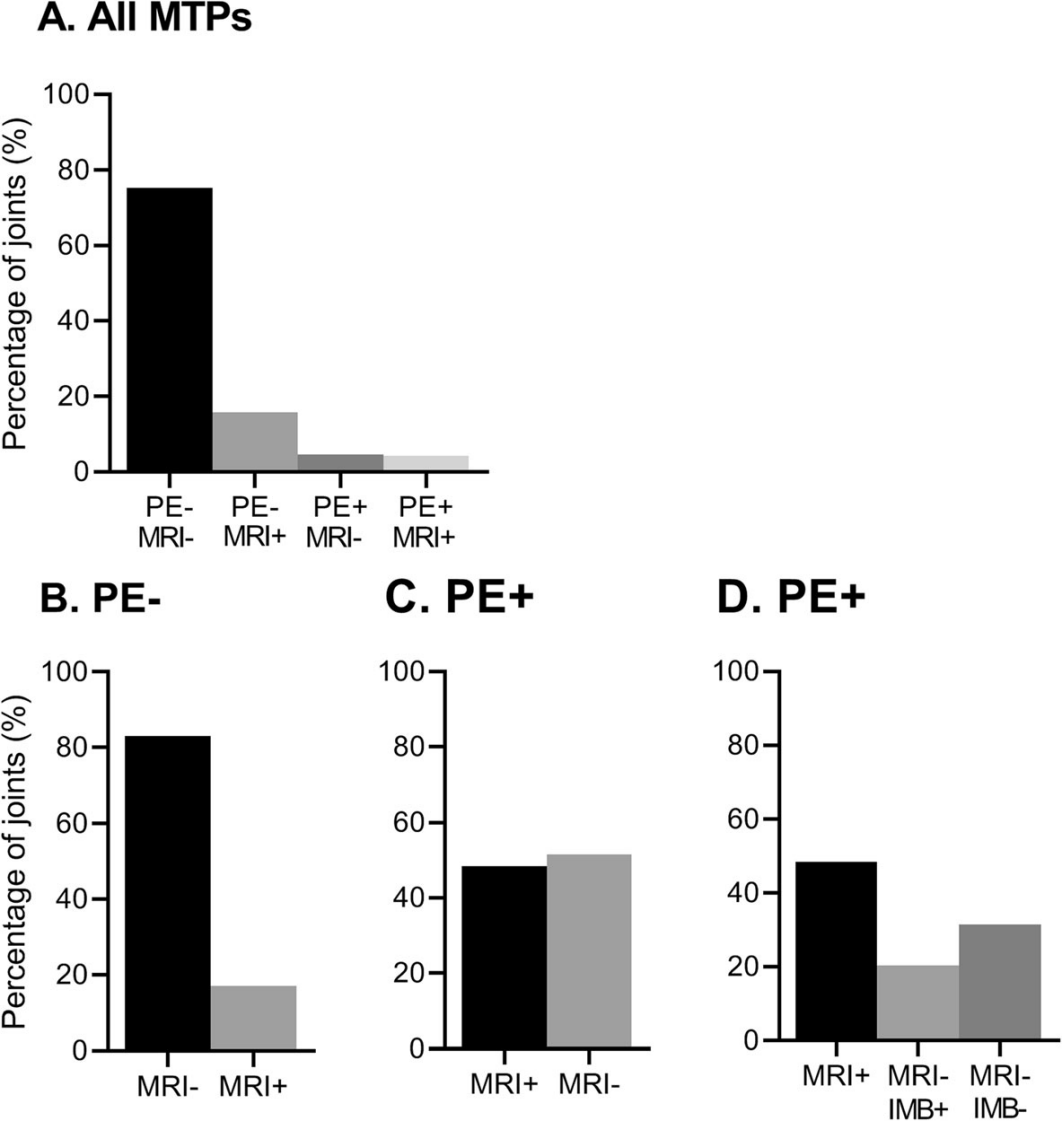
Concordance and discordance between clinical joint swelling at PE and findings at MRI, for all 1759 MTP joints (**a**) and for non-swollen (**b**) and swollen MTP joints (**c**, **d**) separately. The number of joints per group are as follows: PE-MRI− *n* = 1325 joints, PE-MRI+ *n* = 281, PE+MRI− *n* = 79, and PE+MRI+ *n* = 74 joints. **a** Frequency of all 1759 MTP joints per category of PE/MRI. **b** 1606 non-swollen (PE−) MTP joints. **c** 153 swollen (PE+) MTP joints. **d** The same 153 PE+ joints as in **c**; in addition to the frequency of MRI inflammation, the frequency of intermetatarsal bursitis (IMB) is presented in swollen joints that had no MRI inflammation. MTP metatarsophalangeal joints, PE physical joint examination, PE− not swollen, PE+ swollen, MRI magnetic resonance imaging, MRI− absence of MRI-detected inflammation defined as synovitis, tenosynovitis, and osteitis, MRI+ presence of MRI-detected inflammation, IMB intermetatarsal bursitis, either present (+) or absent (−)

Of all 1759 MTP joints, 153 (9%) were clinically swollen (PE positive) and 355 (20%) showed MRI inflammation (MRI positive). Combining the PE and MRI scores revealed that 1325 (75%) of all MTP joints were PE negative/MRI negative (Fig. 2a). The other concordant group was smaller: 73 (4%) of all MTP joints were PE positive/MRI positive. Three hundred sixty joints in total were discordant: of which 281 joints were PE negative/MRI positive (16% of all MTP joints) and 79 were PE positive/MRI negative (5%).

Next, PE-positive and PE-negative joints were studied separately (Fig. 2b, c). Of 1606 PE-negative joints, 83% were MRI negative and 17% were MRI positive, showing subclinical inflammation. Of all 153 PE-positive joints, 48% were MRI positive, whereas 52% (79 joints) were MRI negative.

### Association between synovitis, osteitis, and tenosynovitis and clinical joint swelling

Next, we determined which of the MRI features had the strongest association with joint swelling. In univariable analyses, synovitis, tenosynovitis, and osteitis were all associated with joint swelling (Table 2). As these three features frequently co-occur, a multivariable GEE analysis was performed that included all three variables. Here, only tenosynovitis remained statistically associated with joint swelling (OR 3.19, 95% CI 1.6–6.3).

**Table 2:** Association of MRI-detected inflammation with physical joint examination per type of MRI-detected inflammation (synovitis, tenosynovitis, osteitis) and intermetatarsal bursitis for MTP joints in early arthritis

	Swollen joints	Non-swollen joints	Univariable	Multivariable^1^	Multivariable^2^
	*n* (%)	*n* (%)	OR (95% CI)	OR (95% CI)	OR (95% CI)
Any MRI-detected inflammation*	74 (48)	281 (17)	4.42 (2.9–6.8)		
Synovitis	52 (34)	200 (12)	3.62 (2.3–5.8)	1.68 (0.8–3.5)	1.30 (0.6–2.6)
Tenosynovitis	49 (32)	140 (9)	4.94 (3.0–8.0)	3.19 (1.6–6.3)**	2.21 (1.1–4.3)**
Osteitis	31 (20)	117 (7)	3.20 (1.9–5.5)	1.55 (0.8–3.0)	1.5 (0.8–2.7)
Intermetatarsal bursitis	94 (61)	423 (26)	4.48 (2.8–7.1)		2.91 (1.8–4.8)**

*MRI* magnetic resonance imaging, *MTP* metatarsophalangeal joints, *n* number, *OR* odds ratio, *CI* confidence interval

*Defined by the presence of synovitis, tenosynovitis, and/or osteitis. **Data in italics are statistically significant in multivariable analyses, defined as *P*<0.05

^1^Multivariable model including local synovitis, tenosynovitis, and osteitis

^2^Multivariable model including local synovitis, tenosynovitis, osteitis, and intermetatarsal bursitis

### Relationship of IMB with clinical joint swelling

As we had observed that 52% of swollen MTP joints (79 joints) showed no MRI inflammation, we hypothesized that IMB could explain part of this discordance. IMB was scored in all patients, and the frequency is given in Table 2 and shown per location in Fig. 1. In joints that were negative for MRI inflammation, IMB was more frequent in swollen joints than in non-swollen joints (OR 3.2, 95% CI 1.8–5.7, *P* < 0.001) (Table 3). IMB alone explained 21% of all swollen joints (Fig. 2d).

**Table 3:** Frequency of intermetatarsal bursitis (IMB) according to status at physical joint examination (PE) and MRI for all 1759 MTP joints in early arthritis

	MRI+		MRI−	
	IMB+	IMB−	IMB+	IMB−
PE+, *n* (%)	63 (85)	11 (15)	31 (39)	48 (61)
PE**−**, *n* (%)	177 (63)	104 (37)	245 (18)	1080 (82)

*PE* physical joint examination; *PE+* swollen joints; *PE−* non-swollen joints; *MRI* magnetic resonance imaging; *MRI+* positive for MRI-detected inflammation, defined by the presence of synovitis, tenosynovitis, and/or osteitis; *MRI****−*** negative for MRI-detected inflammation; *IMB* intermetatarsal bursitis, either present (+) or absent (−)

**Fig. 3:**
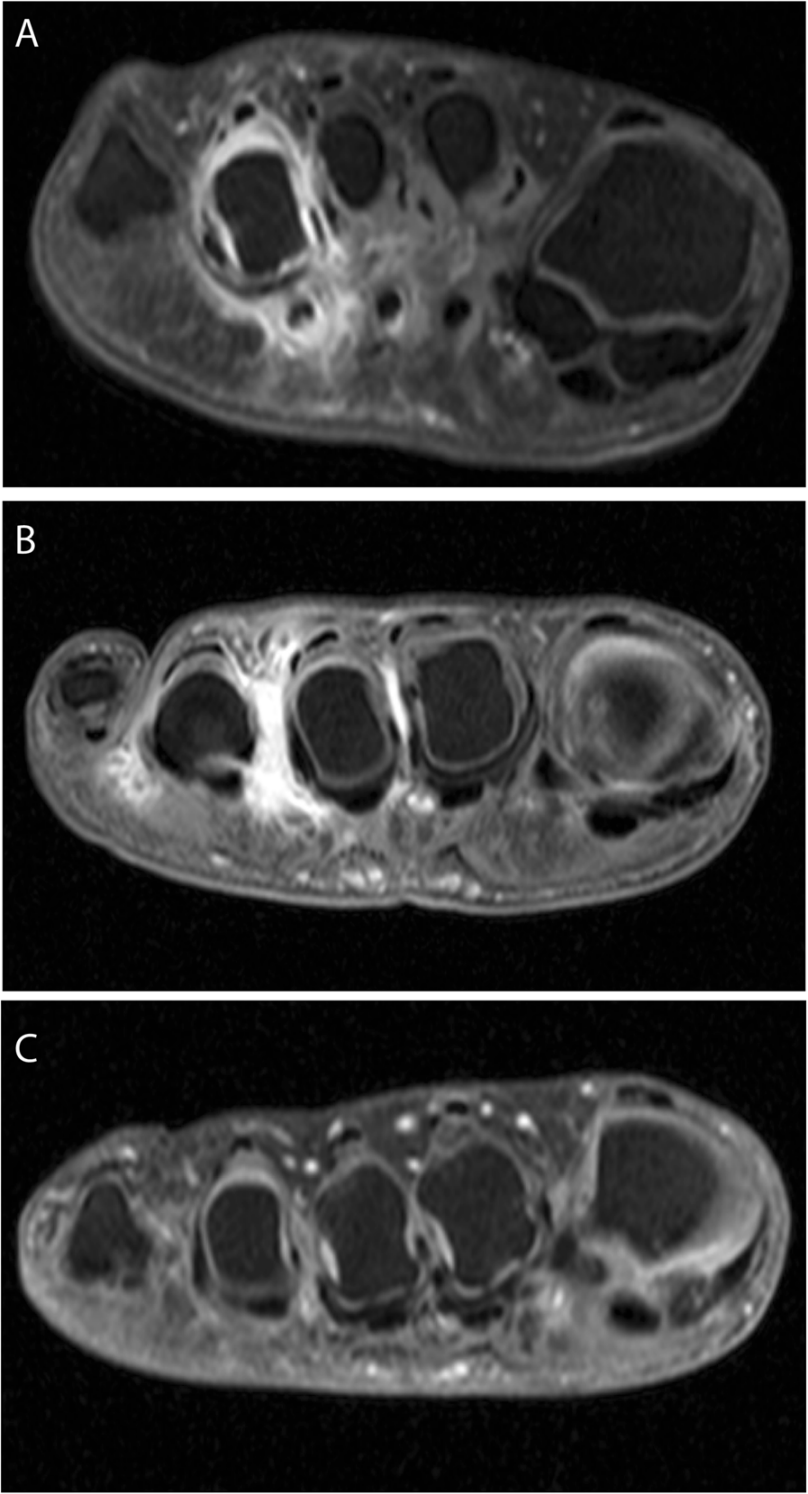
MRI examples of MTP joints that are swollen upon physical joint examination (PE) with MRI-detected inflammation (**a**), with intermetatarsal bursitis (**b**), and without any signs on MRI (**c**). **a** A patient with a swollen MTP 4 upon PE and with MRI-detected synovitis and tenosynovitis at the same joint. **b** A patient with swollen MTP 3 and 4: there is no MRI-detected synovitis, tenosynovitis, or osteitis; there is intermetatarsal bursitis at the 2nd, 3rd, and 4th intermetatarsal spaces. **c** Patient with swollen MTP 2, 3, and 4 upon PE but no MRI-detected inflammation or intermetatarsal bursitis on MRI

When considering all swollen MTP joints (*n* = 153), 48 (31%) could not be explained by MRI-detected inflammation or IMB and thus had no abnormality upon MRI (Fig. 2d). MRI examples of swollen MTP joints are given in Fig. 3.

Thereafter, we included IMB in the analysis assessing the association of MRI findings with clinical joint swelling. Both in univariable and multivariable models, IMB was associated with MTP swelling, next to tenosynovitis (Table 2).

### Concordance and discordance of PE and MRI at MCP joints

The MCP joints were studied for comparison; results are presented in Fig. 1 and Additional file 1: Figure S2. Non-swollen MTP joints showed less subclinical inflammation than non-swollen MCP joints (18 vs. 34%, *P* < 0.001). Swollen MTP joints also showed less MRI inflammation than swollen MCP joints (48% vs. 88%, *P* < 0.001). Finally, at the MCP joints, synovitis was the MRI feature with the strongest association with joint swelling (OR 6.40, 95% CI 4.0–10.2) (Table S1).

### Sensitivity analyses

Analyses were repeated in patients with RA (*n* = 157). Twenty-two percent had ≥ 1 swollen MTP joint (Additional file 1: Figure S3 and S4). Subclinical inflammation was found in 29% of non-swollen MTP joints, and 51% of swollen MTP joints had no MRI inflammation. IMB was found in 54% of these swollen joints without MRI inflammation (Additional file 1: Table S2).

We considered that osteitis and in addition flexor tenosynovitis may not be palpable, considering that PE is performed at the dorsal side of the foot. Therefore, analyses on concordance and discordance were performed excluding osteitis and flexor tenosynovitis from the definition of MRI inflammation. This yielded similar results as the main analyses (Additional file 1: Figure S5).

Finally, non-swollen joints may be tender as a sign of subclinical MRI inflammation. Therefore, analyses were repeated in joints that were tender, and swollen joints were excluded. Of non-tender joints, 85% were MRI negative and 17% were MRI positive. Of tender joints, 33% were MRI positive and 67% MRI negative (Additional file 1: Figure S6). When also considering IMB, tender joints that were unexplained by MRI went from 67 to 43%.

### Some characteristics of patients with swollen MTP joints without MRI findings

As mentioned, 31% of all swollen joints had no MRI abnormality; these belonged to 19 patients. We speculated that these patients may have a higher BMI or possibly peripheral edema. The latter was not scored; therefore, age was used as a rough proxy. Patients with swollen MTP joints without MRI findings had no difference in age or BMI than patients with swollen MTP joints with MRI findings (*n* = 54) (age 54.8 versus 55.0 years, BMI 24.8 versus 25.5).

## Discussion

The MTP joints are a known preferential location of involvement in early arthritis. Nonetheless, in RA outcome measures, they are often ignored [27], as for instance, the Disease Activity Score 28 (DAS28) excludes the feet. This may be due to several reasons, including feasibility, as taking shoes off costs time, but also due to the complexity of elaborate physical examinations [28]. Examination of MTP joints is considered more challenging and perhaps less reliable [6]. As to the best of our knowledge there are no large studies on the relation between joint swelling and findings on MRI in the MTP joints, we performed a large cross-sectional study in early arthritis to increase the understanding of the relationship between swelling upon PE with MRI-detected inflammation of the MTP joints. We found that PE and MRI were mostly in agreement, as most joints were not swollen and had no MRI-detected inflammation. In other words, a negative joint examination largely corresponded to a lack of imaging inflammation. More unexpected, in swollen MTP joints, MRI-detected inflammation was also frequently absent. Thus, while the common notion that PE of MTP joints is difficult is perhaps true, our data revealed that this is not in the direction that most would expect, as our results indicate that PE of MTP joints is perhaps sometimes too sensitive.

Subclinical inflammation was present in 17% of non-swollen MTP joints. The relevance of subclinical inflammation of MTP joints was assessed previously in two studies [29, 30]. The first study analyzed hand and foot joints together, limiting the conclusions that can be drawn regarding the MTP joints [29]. The second study found that osteitis but not synovitis of MTP joints was associated with radiographic progression after 1 year [30]. Tenosynovitis at the MTP joints was not assessed; its relevance for predicting radiographic progression remains a subject for future studies.

We studied IMB to explore whether it can explain part of the observed absence of MRI inflammation in swollen MTP joints. We acknowledge that no validated scoring method exists to score IMB, and scoring was done as part of an exploratory analysis. Therefore, reading was done by consensus rather than by two independent readers. Consensus reading may be necessary in the setting of preliminary findings, when the verification of the general validity of an initial observation is required [31], as was the case in this study. However, for determining the validity of an outcome measure, it is crucial to demonstrate the reliability of scoring between independent readers [31, 32]. Validation of IMB scoring according to the OMERACT Filter would be valuable [32]. Second, further evaluations on IMB in subsequent studies could include the association with RA and within RA with long-term outcomes.

Another pathology in the forefoot is Morton’s neuroma; this lesion originates from the neurovascular bundle and has no synovial lining. It was not included in this study on the relation with PE as it lies plantar to the deep transverse metatarsal ligament and the metatarsal heads [15]. In addition to its location, Morton’s neuroma gives a distinct burning sensation. Therefore, we assumed that clinicians would recognize Morton’s neuroma as a different entity.

Statistically, joint swelling was independently associated with tenosynovitis and IMB in multivariable analyses, in contrast to synovitis. However, tenosynovitis and synovitis often overlap; their collinearity may explain why synovitis was not statistically significant. For the clinician, it may be important to realize that swelling upon PE represents more than just synovitis, but that tenosynovitis and IMB can also be involved.

Surprisingly, 31% of all swollen MTP joints had no MRI abnormalities (Fig. 3b). The origin of this swelling is speculative; possibly subcutaneous tissues (e.g., fat, peripheral edema) underlie the observed swelling. BMI was not associated but is also an incomplete proxy of fat at the forefeet. The joint swelling was also unlikely a simple measurement error as it was observed by two persons independently. Furthermore, joint evaluation was unlikely influenced by laboratory findings as these were not yet performed at the first visit at the outpatient clinic and GPs are discouraged to perform laboratory investigations according to national guidelines [33]. Notably, swollen joints without MRI detected abnormalities occurred more often at MTP joints than MCP joints, despite similar methodology of performing PE and similar scan protocols. All taken into consideration, we cannot explain the pathology underlying swollen MTP joints without MRI abnormality. The clinical relevance of this finding is unclear and needs to be determined in longitudinal studies, specifically if long-term outcomes are different between swollen joints with and without MRI abnormalities.

An important limitation of this study is the reproducibility of PE. Determination of joint swelling is notoriously difficult and poorly reproducible [6, 34]. Variation between readers can be reduced by regular consensus exercises for swollen joint assessment [35]; such sessions were regularly held at our department. To further reduce reader dependency, we applied a stringent definition for a swollen joint, as it had to be observed by two assessors that independently evaluated the patient the same day. This increased the certainty of the presence of the feature, by reducing the chance of ‘overscoring’ any doubtfully swollen MTP joint.

Deliberately, MTP1 was not included as it is also a predilection site for disease other than early arthritis, e.g., osteoarthritis and gout. As these degenerative diseases can also give MRI-detected inflammation that is not necessarily related to arthritis, MTP1 was excluded as it could hamper the comparison between findings on PE and MRI.

Disease activity is assessed by composite indices, like the DAS [36]. However, for many treating physicians, the presence of swollen joints may carry substantial weight, regardless of whether a patient is in DAS remission. MRI-detected inflammation is associated with radiographic progression [29], and the assumption is that MRI-detected inflammation underlies joint swelling. The question rises whether swelling without underlying MRI inflammation is clinically relevant, i.e., whether it leads to radiographic progression. Longitudinal studies are needed to determine the clinical relevance of MTP swelling without inflammation, for instance by comparing radiographic progression of swollen MTP joints with and without MRI-detected inflammation.

## Conclusion

This is the largest study to date to compare PE with MRI in consecutively presenting early arthritis patients. A negative PE of the MTP joints is reassuring, as MRI-detected subclinical inflammation is most often absent. Joint swelling of MTP joints is traditionally interpreted as synovitis; we showed that pathologies underlying joint swelling at this location also include tenosynovitis and IMB, and finally, MTP swelling remained unexplained by MRI in part of the joints. Validation studies are warranted. Subsequently longitudinal studies are required to determine the clinical relevance of joint swelling without underlying MRI-detected inflammation.

## Supplementary information

**Additional file 1: Supplementary Methods 1**. Detailed MRI protocol. **Supplementary Methods 2**. Scoring of MRI-inflammation: synovitis, tenosynovitis and osteitis. **Table S1**. Association of MRI-detected inflammation with physical joint examination per type of MRI-detected lesion (synovitis, tenosynovitis and osteitis) for MCP-joints. **Table S2**. Frequency of intermetatarsal bursitis (IMB) according to status at physical joint examination (PE) and MRI in MTP-joints in 157 RA-patients. **Figure S1**. Schematic illustration with coronal view of the forefoot at the metatarsal heads (M1–5) with intermetatarsal bursae in between. **Figure S2**. Concordance and discordance between clinical joint swelling at PE and findings at MRI, for all 1750 MCP-joints (A) and for non-swollen (B) and swollen MCP-joints (C) separately. **Figure S3**. Frequency of a swollen joint upon physical examination, MRI-detected inflammation and intermetatarsal bursitis (IMB) per location for MTP(2–5)- and MCP(2–5)-joints in 157 RA patients. **Figure S4**. Concordance and discordance between clinical joint swelling at PE and findings at MRI, for all 625 MTP-joints (A) and for non-swollen (B) and swollen MTP-joints (C, D) separately in 157 RA-patients. **Figure S5**. Concordance and discordance between clinical joint swelling at PE and findings at MRI, for all 1759 MTP-joints (A) and for non-swollen (B) and swollen MTP-joints (C, D) separately when flexor tenosynovitis and osteitis were not included in definition of MRI-inflammation. **Figure S6**. Concordance and discordance between joint tenderness at PE and findings at MRI, excluding swollen joints, given for all MTP-joints (A) and for non-tender (B) and tender MTP-joints separately (C, D).

## Data Availability

The datasets used and/or analyzed during the current study are available from the corresponding author on reasonable request.
