# Supplementary material for: The relation between physical joint examination and MRI-depicted inflammation of metatarsophalangeal joints in early arthritis
Source: Arthritis Res Ther. 2020 Apr 3;22:67. doi: 10.1186/s13075-020-02162-7 (PMC7118815; doi:10.1186/s13075-020-02162-7)
Supplement: Supplementary file 1 — Additional file 1: Supplementary Methods 1. Detailed MRI protocol. Supplementary Methods 2. Scoring of MRI-inflammation: synovitis, tenosynovitis and osteitis. Table S1. Association of MRI-detected inflammation with physical joint examination per type of MRI-detected lesion (synovitis, tenosynovitis and osteitis) for MCP-joints. Table S2. Frequency of intermetatarsal bursitis (IMB) according to status at physical joint examination (PE) and MRI in MTP-joints in 157 RA-patients. Figure S1. Schematic illustration with coronal view of the forefoot at the metatarsal heads (M1–5) with intermetatarsal bursae in between. Figure S2. Concordance and discordance between clinical joint swelling at PE and findings at MRI, for all 1750 MCP-joints (A) and for non-swollen (B) and swollen MCP-joints (C) separately. Figure S3. Frequency of a swollen joint upon physical examination, MRI-detected inflammation and intermetatarsal bursitis (IMB) per location for MTP(2–5)- and MCP(2–5)-joints in 157 RA patients. Figure S4. Concordance and discordance between clinical joint swelling at PE and findings at MRI, for all 625 MTP-joints (A) and for non-swollen (B) and swollen MTP-joints (C, D) separately in 157 RA-patients. Figure S5. Concordance and discordance between clinical joint swelling at PE and findings at MRI, for all 1759 MTP-joints (A) and for non-swollen (B) and swollen MTP-joints (C, D) separately when flexor tenosynovitis and osteitis were not included in definition of MRI-inflammation. Figure S6. Concordance and discordance between joint tenderness at PE and findings at MRI, excluding swollen joints, given for all MTP-joints (A) and for non-tender (B) and tender MTP-joints separately (C, D). [file 13075_2020_2162_MOESM1_ESM.docx]

**Additional File 1**

# Supplementary Methods 1: Detailed MRI protocol

MR imaging was performed on a MSK-extreme 1.5T extremity MR imaging system (GE, Wisconsin, USA) using a 145mm coil for the foot and a 100mm coil for the hand. The patient was positioned in a chair beside the scanner, with the hand or foot fixed in the coil with cushions.

In the hand (metacarpophalangeal-(MCP) joints) the following sequence was acquired before contrast administration: T1-weighted fast spin-echo (FSE) sequence in the coronal plane (repetition time (TR) 575 ms, echo time (TE) 11.2 ms, acquisition matrix 388×288, echo train length (ETL) 2). After intravenous injection of gadolinium contrast (gadoteric acid, Guerbet, Paris, France, standard dose of 0.1 mmol/kg) the following sequences were obtained: T1-weighted FSE sequence with frequency selective fat saturation (fatsat) in the coronal plane (TR/TE 700/9.7ms, acquisition matrix 364×224, ETL 2), T1-weighted FSE sequence with frequency selective fat saturation in the axial plane (TR/TE 570/7.7 ms; acquisition matrix 320x192; ETL 2).

The following sequences of the forefoot (metatarsophalangeal-(MTP) joints) were obtained after intravenous injection of gadolinium contrast: T1-weighted FSE fatsat sequence in the axial plane (TR/TE 700/9.5ms; acquisition matrix 364x224, ETL 2) and: T1-weighted FSE fatsat sequence in the coronal plane (perpendicular to the axis of the MTP-joints) (TR/TE 540/7.5ms; acquisition matrix 320x192, ETL 2). Field-of-view was 100mm for the hand and 140mm for the foot. Coronal sequences of the hand had 18 slices with a slice thickness of 2mm and a slice gap of 0.2mm. Coronal sequences of the foot had 20 slices with a slice thickness of 3mm and a slice gap of 0.3mm. All axial sequences had a slice thickness of 3mm and a slice gap of 0.3mm with 16 for the metacarpophalangeal-joints and 14 for the foot.

According to the RAMRIS-method, T2-weighted fat suppressed or short tau inversion recovery (STIR) sequences should be used to assess osteitis. Previously, three studies have demonstrated that a contrast enhanced T1-weigthed fat suppressed sequence has a strong correlation with T2-weighted fat suppressed sequences.[1-3] The European Society of Musculoskeletal Radiology (ESSR) Arthritis Subcommittee also recommends the use of contrast enhanced T1-weighted fat suppressed sequences for depiction of osteitis.[4] We used the contrast enhanced T1-weighted fat suppressed sequence as it allowed a shorter scan time and has a higher signal to noise ratio.

# Supplementary Methods 2: Scoring of MRI-inflammation: synovitis, tenosynovitis and osteitis

Synovitis and osteitis at MTP- and MCP-joints were scored in line with the validated Outcome Measures in Rheumatology Clinical Trials Rheumatoid Arthritis Magnetic Resonance Imaging Scoring-system (OMERACT RAMRIS).[5] For tenosynovitis, the score as described by Havaardsholm *et al* was applied to the extensor and flexor tendons of the MTP- and MCP-joints.[6]

The synovitis score (range 0-3) was scored based on the volume of enhancing tissue in the synovial compartment (none, mild, moderate, severe), the tenosynovitis-score (range 0-3) was based on the thickness of peritendinous effusion or synovial proliferation with contrast enhancement (normal, <2mm, 2-5mm, >5mm) and osteitis was scored on a 0-3 scale based on the affected volume of the bone (no osteitis, >0-33%, >33-66%, >66%).

The scores of proximal and distal osteitis were summed into osteitis scores and scores of flexor and extensor tenosynovitis into tenosynovitis scores.

Missing scores: The MRI values of 19 out of 3528 joints (5 MTP- and 14 MCP-joints) were missing as due to movement artefacts or inhomogeneous fat suppression these joints could not be scored. None of these 5 MTP-joints were swollen upon PE, 5 out of the 14 MCP-joints were swollen. As joint-level analyses were performed, these values were not imputed.

# Table S1. Association of MRI-detected inflammation with physical joint examination per type of MRI-detected lesion (synovitis, tenosynovitis and osteitis) for MCP-joints

|  | Swollen joints | Non-swollen joints | Univariable | Multivariable¹ |
| --- | --- | --- | --- | --- |
|  | **n (%)** | **n (%)** | **OR (95% CI)** | **OR (95% CI)** |
| Any MRI-detected inflammation* | 199 (88) | 516 (34) | 14.40 (9.2-22.6) |  |
|  |  |  |  |  |
| Synovitis | 184 (81) | 395 (26) | 12.50 (9.6-18.2) | 6.40 (4.0-10.2) |
| Tenosynovitis | 152 (67) | 331 (22) | 7.40 (5.3-10.4) | 2.56 (1.7-3.9) |
| Osteitis | 53 (24) | 72 (5) | 6.19 (4.1-9.4) | 2.81 (1.8-4.3) |

MRI: magnetic resonance imaging; MCP: metacarpophalangeal joints; n: number; OR: odds ratio, CI: confidence interval.

*Defined by the presence of synovitis, tenosynovitis and/or osteitis.

¹Multivariable model including local synovitis, tenosynovitis and osteitis.

# **Table S2**. Frequency of intermetatarsal bursitis (IMB) according to status at physical joint examination (PE) and MRI in MTP-joints in 157 RA-patients

|  | MRI + | | MRI - | |
| --- | --- | --- | --- | --- |
|  | **IMB+** | **IMB-** | **IMB+** | **IMB-** |
| PE +, n (%) | 37 (93) | 3 (8) | 22 (54) | 19 (46) |
| PE -, n (%) | 113 (72) | 44 (28) | 138 (36) | 249 (64) |

PE: physical joint examination: PE+: swollen joints; PE-: non-swollen joints; MRI: magnetic resonance imaging; MRI+: positive for MRI-detected inflammation, defined by the presence of synovitis, tenosynovitis and/or osteitis; MRI-: negative for MRI-detected inflammation; IMB: intermetatarsal bursitis, either present (+) or absent (-).

# Figure S1. Schematic illustration with coronal view of the forefoot at the metatarsal heads (M1-5) with intermetatarsal bursae in between


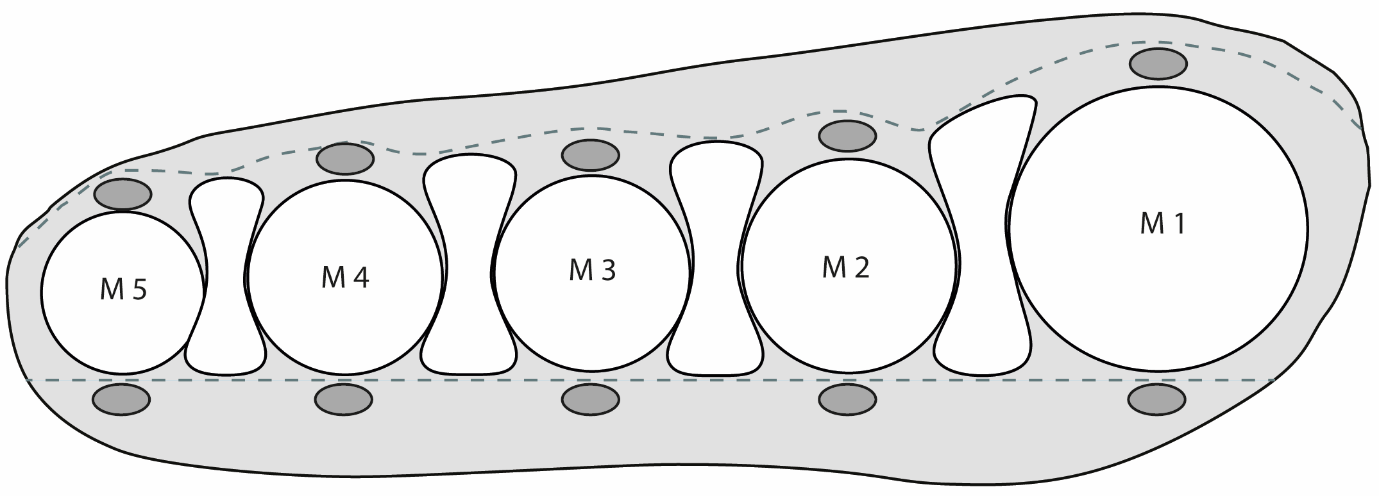


The intermetatarsal bursae are located between the metatarsal heads (M1-5). They are bound dorsally by the dorsal aponeurosis (dashed line dorsal of bursae) and plantarly by the deep transverse metatarsal ligament (dashed line plantar of bursae). Grey ovals represent the extensor and flexor tendons.

# Figure S2. Concordance and discordance between clinical joint swelling at PE and findings at MRI, for all 1750 MCP-joints (A) and for non-swollen (B) and swollen MCP-joints (C) separately


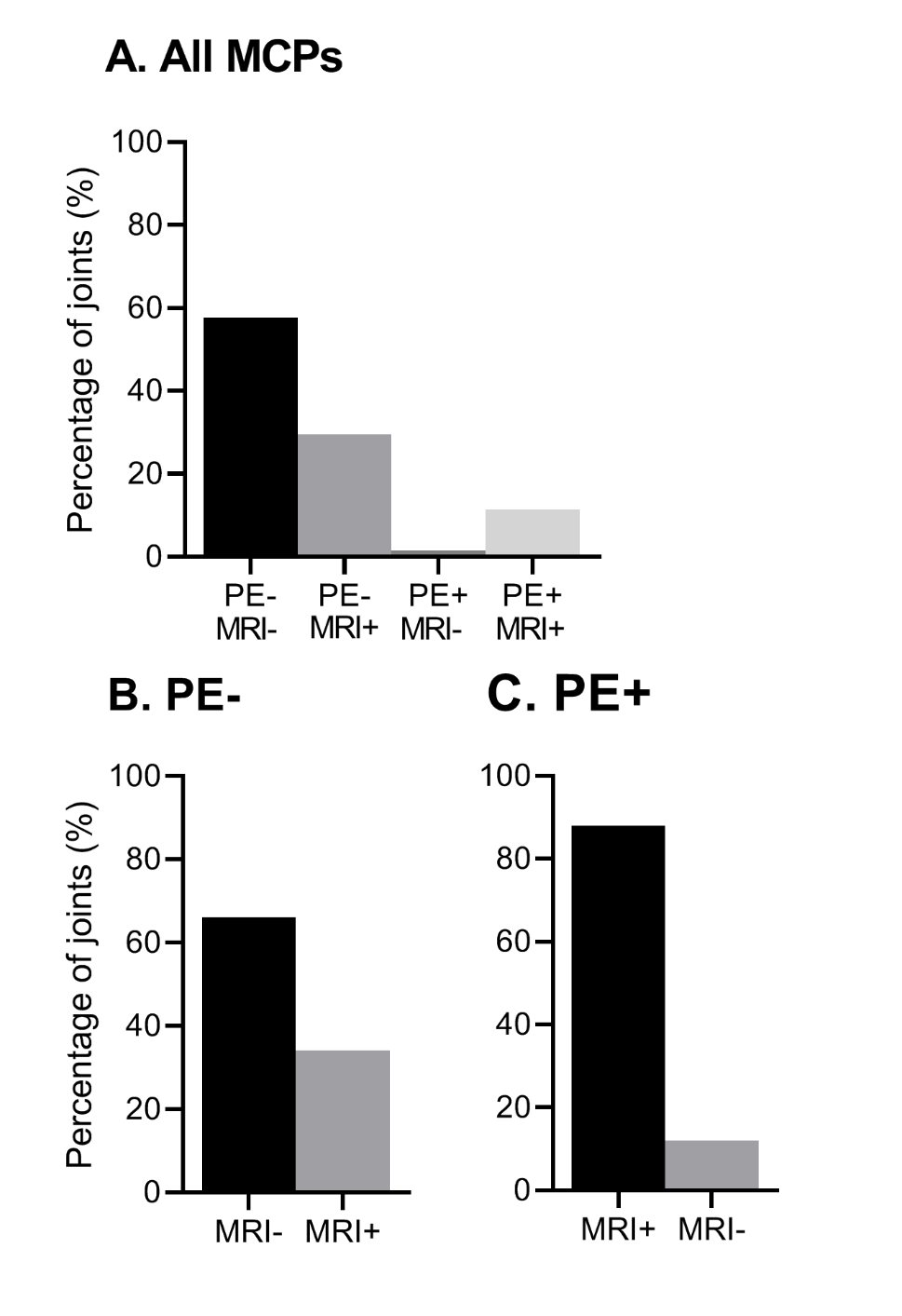


MCP: metacarpophalangeal joints; PE: physical joint examination; MRI: magnetic resonance imaging; MRI-: absence of MRI-detected inflammation defined as synovitis, tenosynovitis and osteitis; MRI+: absence of MRI-detected inflammation. The percentage of joints in each group are illustrated in the figure, the number of joint per group are as follows; PE-MRI- are n=1008 joints, PE-MRI+ n=516, PE+MRI- n=27 and PE+MRI+ n=199 joints.

# Figure S3. Frequency of a swollen joint upon physical examination, MRI-detected inflammation and intermetatarsal bursitis (IMB) per location for MTP(2-5)- and MCP(2-5)-joints in 157 RA patients


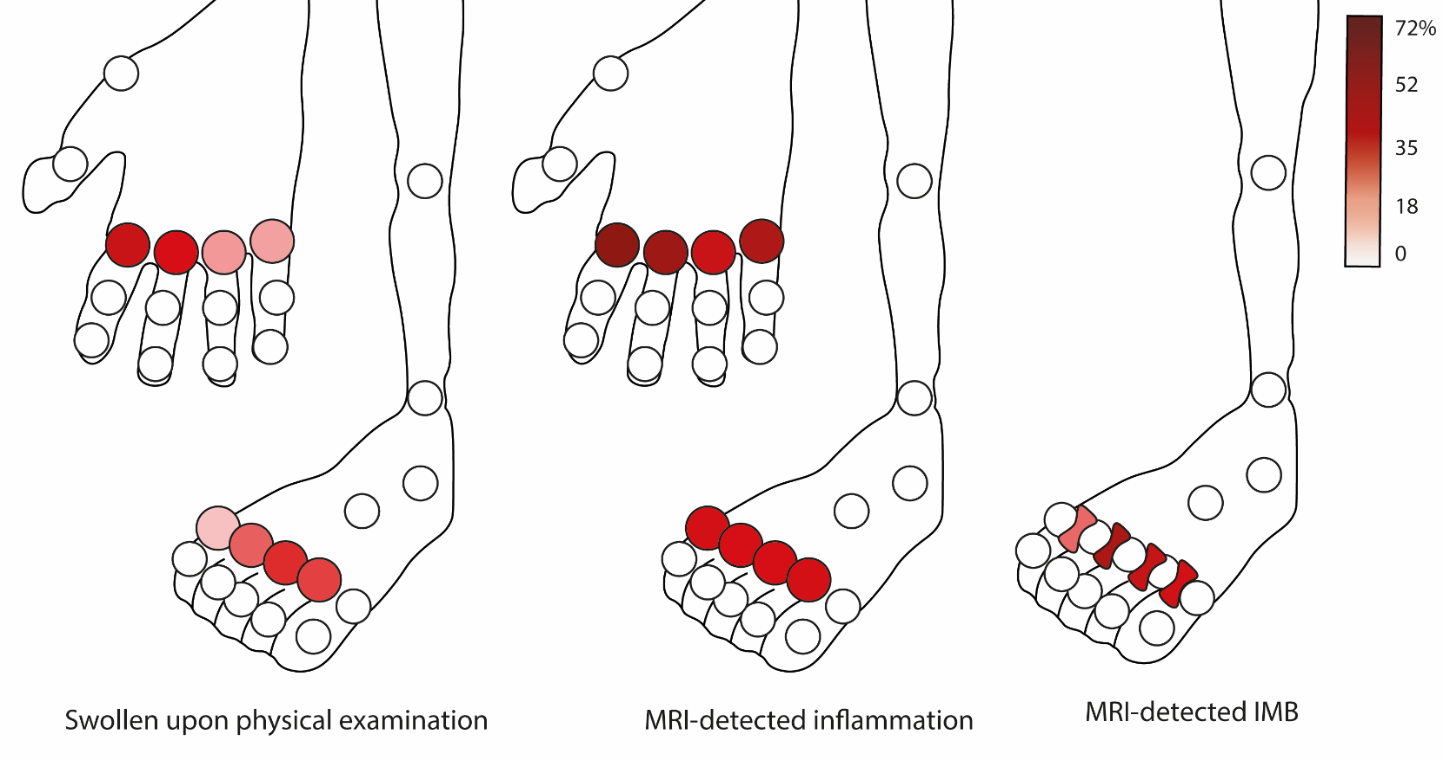


RA was defined according to the clinical diagnosis and fulfilling the 2010 criteria during the first year of follow-up

# **Figure S4.** Concordance and discordance between clinical joint swelling at PE and findings at MRI, for all 625 MTP-joints (A) and for non-swollen (B) and swollen MTP-joints (C, D) separately in 157 RA-patients


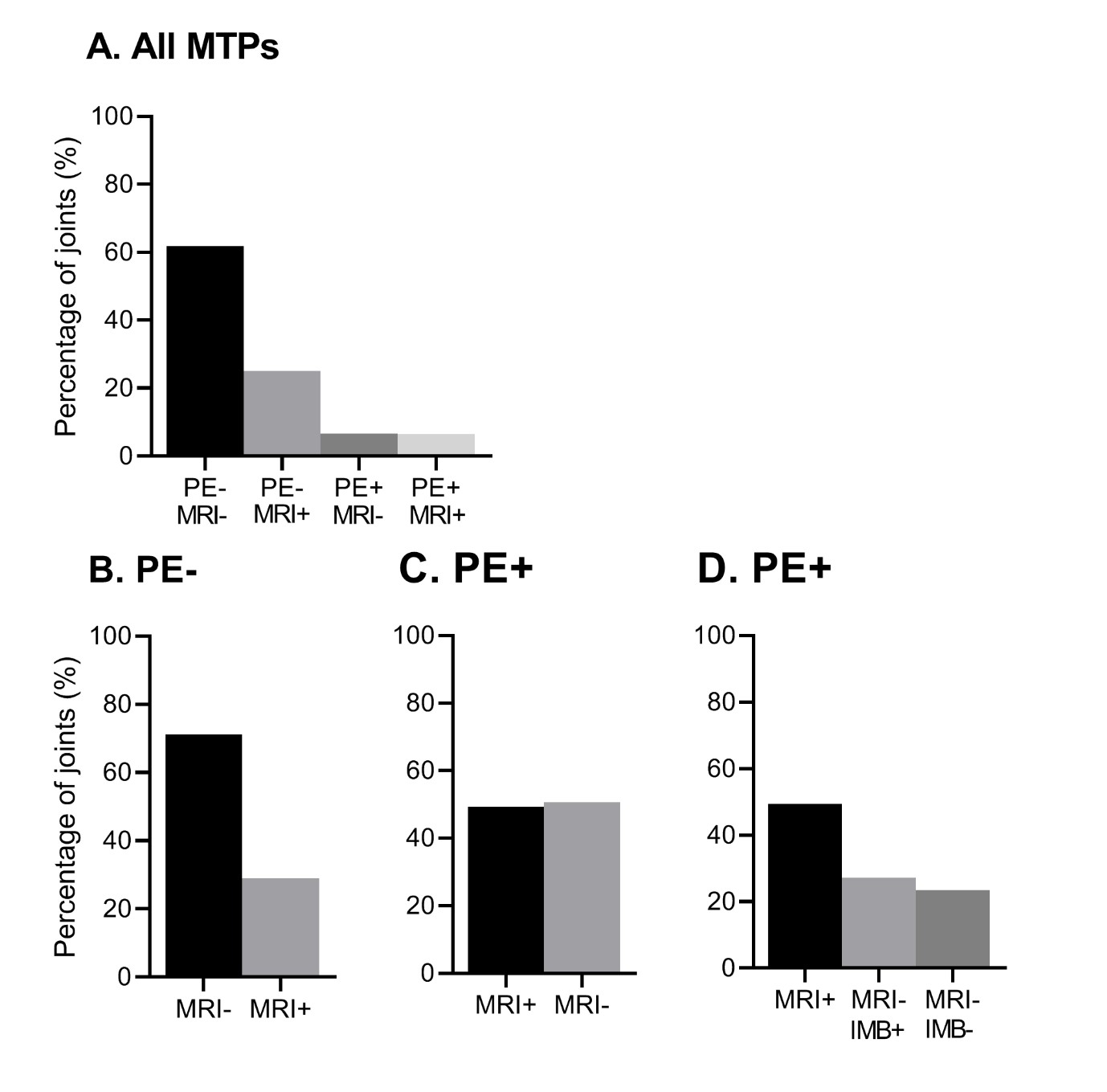


MTP: metatarsophalangeal joints; PE: physical joint examination; PE-: not swollen; PE+: swollen; MRI: magnetic resonance imaging; MRI-: absence of MRI-detected inflammation defined as synovitis, tenosynovitis and osteitis; MRI+: absence of MRI-detected inflammation; IMB: intermetatarsal bursitis, either present (+) or absent (-). RA was defined according to the clinical diagnosis and fulfilling the 2010 criteria during the first year of follow-up.

# **Figure S5.** Concordance and discordance between clinical joint swelling at PE and findings at MRI, for all 1759 MTP-joints (A) and for non-swollen (B) and swollen MTP-joints (C, D) separately when flexor tenosynovitis and osteitis **were not included** in definition of MRI-inflammation


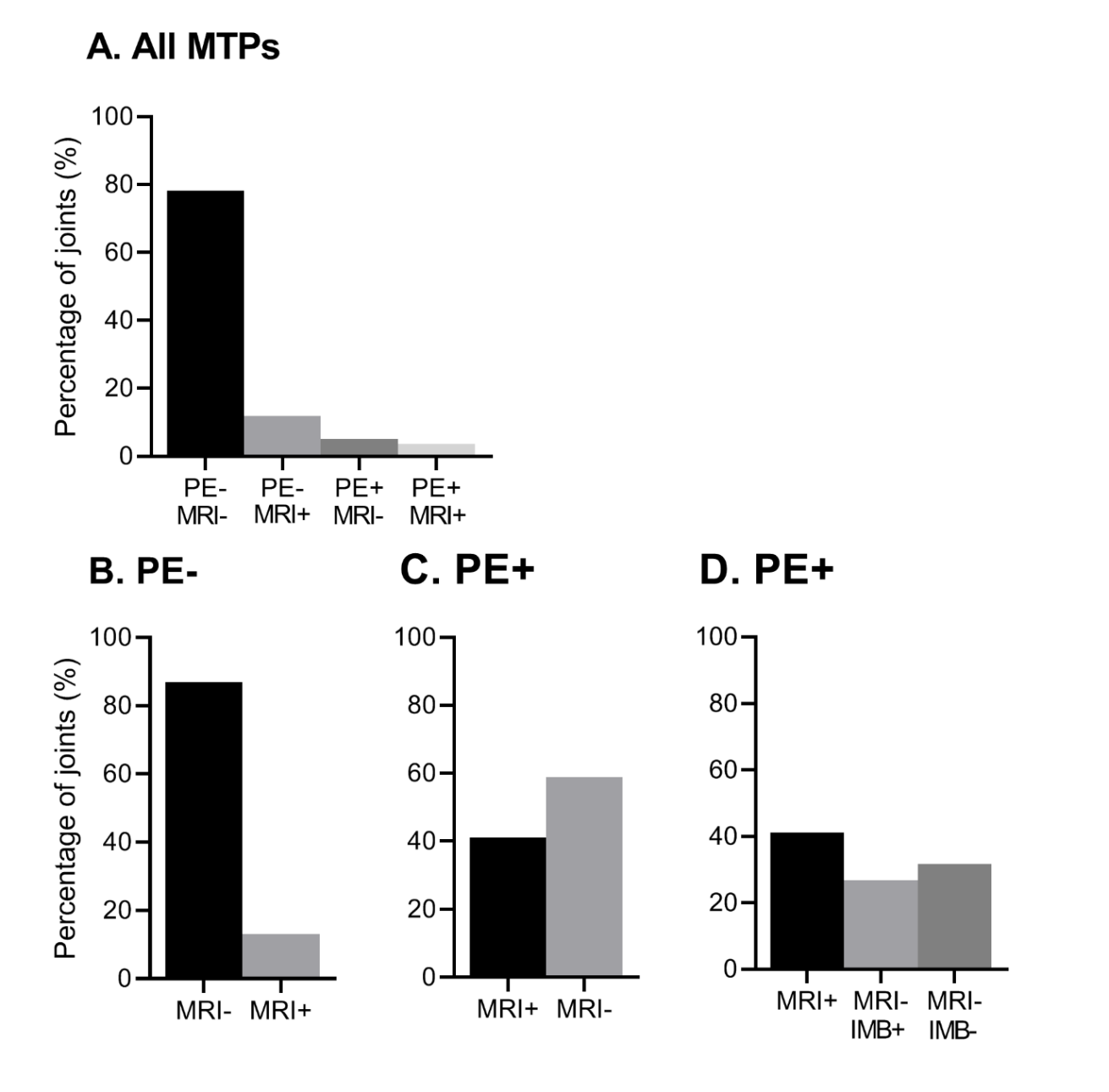


MTP: metatarsophalangeal joints; PE: physical joint examination; PE-: not swollen; PE+: swollen; MRI: magnetic resonance imaging; MRI-: absence of MRI-detected inflammation defined as synovitis and/or extensor tenosynovitis; MRI+: absence of MRI-detected inflammation; IMB: intermetatarsal bursitis, either present (+) or absent (-).

# **Figure S6.** Concordance and discordance between joint tenderness at PE and findings at MRI, excluding swollen joints, given for all MTP-joints (A) and for non-tender (B) and tender MTP-joints separately (C, D)


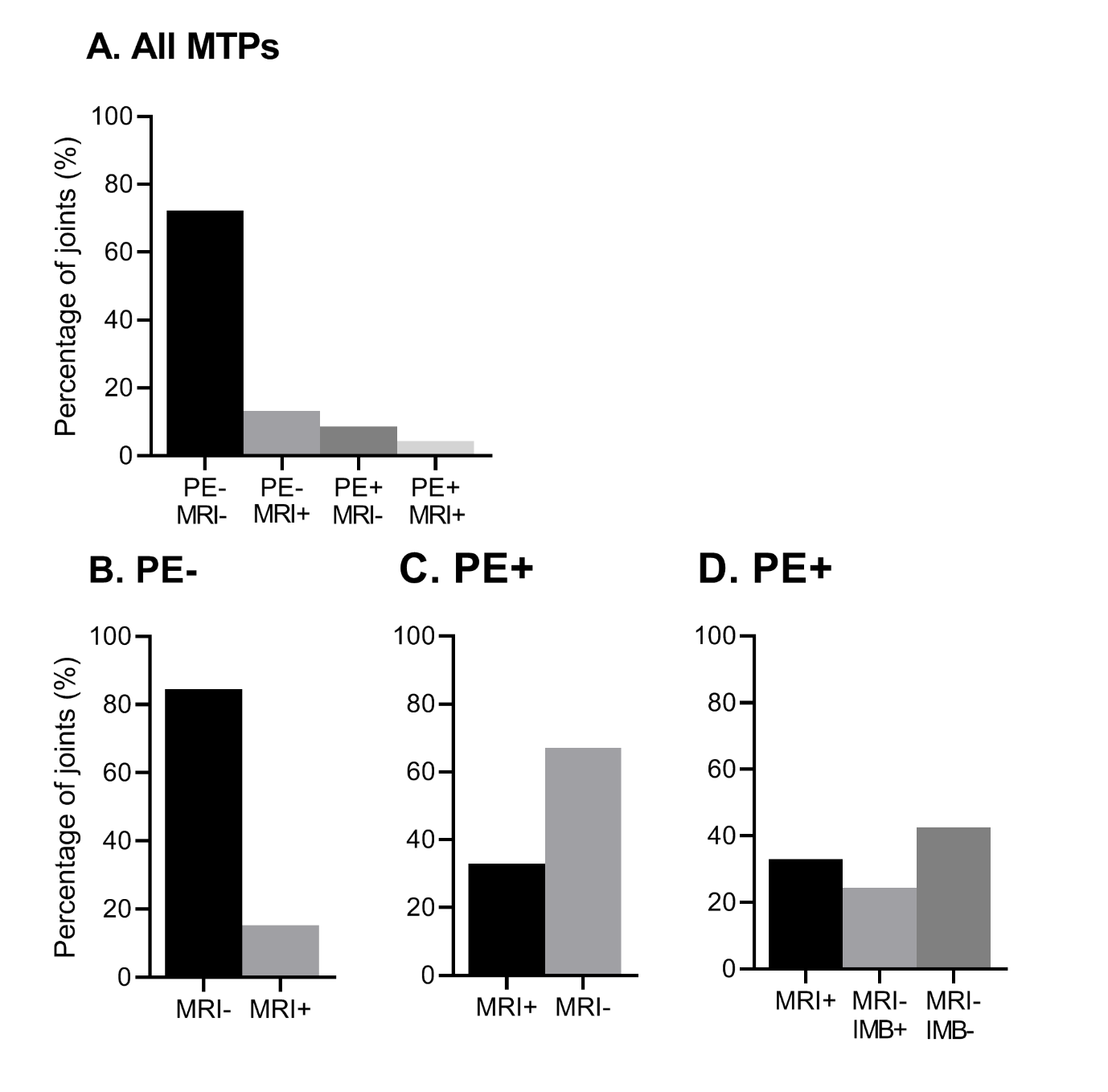


PE+ defined as tender upon physical joint examination. Swollen joints (n= 153) were excluded from this analysis. Tender joints were evaluated by one assessor.

# REFERENCES

1. Mayerhoefer ME, Breitenseher MJ, Kramer J*, et al.* STIR vs. T1-weighted fat-suppressed gadolinium-enhanced MRI of bone marrow edema of the knee: computer-assisted quantitative comparison and influence of injected contrast media volume and acquisition parameters. J Magn Reson Imaging. 2005 Dec; 22:788-93.

2. Schmid MR, Hodler J, Vienne P*, et al.* Bone marrow abnormalities of foot and ankle: STIR versus T1-weighted contrast-enhanced fat-suppressed spin-echo MR imaging. Radiology. 2002 Aug; 224:463-9.

3. Stomp W, Krabben A, van der Heijde D*, et al.* Aiming for a shorter rheumatoid arthritis MRI protocol: can contrast-enhanced MRI replace T2 for the detection of bone marrow oedema? Eur Radiol. 2014 Oct; 24:2614-22.

4. Sudol-Szopinska I, Jurik AG, Eshed I*, et al.* Recommendations of the ESSR Arthritis Subcommittee for the Use of Magnetic Resonance Imaging in Musculoskeletal Rheumatic Diseases. Semin Musculoskelet Radiol. 2015 Sep; 19:396-411.

5. Ostergaard M, Edmonds J, McQueen F*, et al.* An introduction to the EULAR-OMERACT rheumatoid arthritis MRI reference image atlas. Ann Rheum Dis. 2005 Feb; 64 Suppl 1:i3-7.

6. Haavardsholm EA, Ostergaard M, Ejbjerg BJ*, et al.* Introduction of a novel magnetic resonance imaging tenosynovitis score for rheumatoid arthritis: reliability in a multireader longitudinal study. Ann Rheum Dis. 2007 Sep; 66:1216-20.
